# Ten-Year Trends in Clinical Profiles, Management, and Outcomes of De Novo Acute Heart Failure

**DOI:** 10.3390/jcm15031194

**Published:** 2026-02-03

**Authors:** Francisco Ruiz-Ruiz, Patricia Rodríguez-Torres, Asunción Navarro-Puerto, David Lora-Pablos, Miguel Menéndez-Orenga, Juan Manuel Guerra-Vales, Luis Gómez-Morales, Enrique J. Calderón, Francisco J. Medrano

**Affiliations:** 1 Servicio de Medicina Interna, Hospital San Juan de Dios del Aljarafe, 41930 Bormujos, Spain; francisruizruiz@hotmail.com (F.R.-R.); lgm1983sevilla@gmail.com (L.G.-M.); 2 Servicio de Medicina Interna, Hospital Universitario de Valme, 41014 Sevilla, Spain; patricia.rtorres@hotmail.com (P.R.-T.); masunnp@hotmail.com (A.N.-P.); 3 Unidad de Investigación Clínica, Hospital 12 de Octubre, 28041 Madrid, Spain; david@h12o.es (D.L.-P.); miguelmenendez.imas12@h12o.es (M.M.-O.); 4 Servicio de Medicina Interna, Hospital 12 de Octubre, 28041 Madrid, Spain; fruiz150487@gmail.com; 5 Servicio de Medicina Interna, Hospital Universitario Virgen del Rocío (HUVR), 41013 Sevilla, Spain; ecalderon@us.es; 6 Instituto de Biomedicina de Sevilla, IBIS (Universidad de Sevilla, HUVR, Junta de Andalucía, CSIC), 41013 Sevilla, Spain; 7 CIBER de Epidemiología y Salud Pública (CIBERESP), 28029 Madrid, Spain

**Keywords:** heart failure, prognosis, ten-year trends, multimorbidity, clinical phenotypes, internal medicine, adherence to guidelines, elderly patients, real-world evidence

## Abstract

**Objective:** Heart failure (HF) remains a major global health challenge. We evaluated ten-year trends in clinical profiles, diagnostic/therapeutic management, and outcomes in patients hospitalized for de novo acute heart failure (AHF). **Methods:** We compared two multicenter cohorts of patients admitted to Internal Medicine departments in Spain for a first episode of HF (excluding acutely decompensated chronic HF): a retrospective cohort (CH-2005; *n* = 600) and a prospective cohort (CH-2015; *n* = 180). We assessed clinical characteristics, adherence to guideline-recommended diagnostic testing, discharge treatment, and 12-month outcomes (HF readmissions and all-cause mortality). **Results:** The patients in CH-2015 showed a markedly higher comorbidity burden (Charlson Comorbidity Index > 2: 90.0% vs. 12.8%, *p* < 0.001) and higher chronic kidney disease prevalence (17.8% vs. 11.8%, *p* = 0.01), while mean age was similar (75.0 vs. 73.6 years, *p* = 0.16). Diagnostic adherence improved with higher echocardiography use (92.2% vs. 66.5%, *p* < 0.001). Discharge beta-blocker prescriptions increased (50.6% vs. 31.3%, *p* < 0.001). HF readmissions were higher in CH-2015 at 1 month (35.6% vs. 7.3%, *p* < 0.001) and 12 months (49.4% vs. 21.2%, *p* < 0.001). One-year mortality was higher in CH-2015 (22.5% vs. 16.3%, *p* = 0.07). In the multivariable analyses, the between-cohort difference in mortality was not significant, whereas the higher readmission risk in CH-2015 persisted. **Conclusions:** Over a decade, de novo AHF admissions shifted toward a more complex, multimorbid phenotype. Despite improved diagnostic testing and small advances in evidence-based therapy, the readmission burden increased, highlighting the need for integrated, post-discharge management strategies in older multimorbid populations.

## 1. Introduction

Heart failure (HF) remains a primary global health challenge, with recent estimates showing a continuous rise in prevalence and years lived with disability, particularly in aging populations [1]. In Spain, it is the leading cause of hospitalization in people over 65 years old. It accounts for 3% of all hospital admissions and 2.5% of healthcare costs [2], with HF decompensation hospitalizations accounting for more than 50% of healthcare costs [3]—a figure that has been increasing over the last five years [4]. Moreover, as a consequence of the progressive aging of the population and the increase in cardiovascular diseases and risk factors, hospital admissions for HF in Spain rose by more than 50% between 2007 and 2017 [5]. In Spain, the in-hospital mortality rate for HF is 9.4% [4] and the readmission rate is 9.7% [5]. HF has a one-year mortality rate of 23.6% according to recent registries from European and Mediterranean countries [6].

There is significant variability in the clinical management of HF [7,8], and there are no registries in Spain that directly analyze its evolution over time or the impact of changes in the clinical profile of patients on disease prognosis.

The objectives of the study were to describe, in our setting, the longitudinal trends in the clinical characteristics and comorbidities of patients hospitalized for a first episode of HF over a period of a decade; to identify changes in the adherence to diagnostic and therapeutic guidelines; and to describe the impact of changes in the clinical spectrum and management of HF on the prognosis of the disease (HF readmissions and all-cause mortality) during the first year of follow-up after hospitalization.

## 2. Materials and Methods

### 2.1. Design and Setting

This is a multicenter observational study that included two cohorts of patients with no prior history of heart failure (HF) who were admitted for a first episode of HF in three university hospitals (Virgen del Rocío and Virgen de Valme in Seville, and 12 de Octubre in Madrid), who were used to develop and validate the PREDICE prognostic scale [9,10].

### 2.2. Sample Size Calculation

The sample size of the two cohorts was determined using the PREDICE score validation framework and was primarily aimed at ensuring adequate precision for estimating key clinical characteristics and adherence-to-care indicators rather than detecting small-to-moderate differences in outcomes such as 12-month mortality; therefore, mortality comparisons should be interpreted with caution and considering the effect sizes.

For the first retrospective cohort, based on an estimated variability in the adequacy of drug and procedure use of 25% [9], with a precision of 3%, a confidence level of 95%, and an estimated dropout rate of 10%, the calculated sample size was 600 [10]. For the second prospective cohort, considering the results of the first study, a variability of 5% was estimated using the same alpha and beta error levels, resulting in a calculated sample size of 180 [11].

### 2.3. Study Population

The inclusion criteria were as follows: age > 18 years; no previous history of HF; HF diagnosis according to the Framingham criteria as the primary diagnosis for hospitalization during the study period; and residence within the hospital’s catchment area.

A total of 780 patients hospitalized for a first episode of HF were included in the study, who were distributed across two cohorts:

Retrospective 2005 cohort (CH-2005): 600 patients, 200 per center between 2003 and 2006.

Prospective 2015 cohort (CH-2015): 180 patients, 60 per center between 2013 and 2015.

Participants were enrolled by simple random sampling based on clinical judgment at admission; the cases in which the diagnosis of HF was not confirmed at discharge were excluded.

De novo HF was defined as a first hospitalization for HF in patients without a prior documented HF diagnosis and without previous HF hospitalizations.

### 2.4. Study Variables

The study variables were age, sex, independence in basic activities of daily living (BADL), comorbidities (diabetes mellitus (DM), ischemic heart disease (IHD), atrial fibrillation, valvular heart disease, peripheral arterial disease (PAD), chronic kidney disease (CKD), chronic obstructive pulmonary disease (COPD), and Charlson comorbidity Index (CCI)) [12], diagnostic test results at admission (plasma levels of N-terminal pro-B-type natriuretic peptide (NT-proBNP) and transthoracic echocardiography (TTE)), pharmacological treatment at discharge, and outcomes (HF readmissions and all-cause mortality at one year post-discharge). HF readmissions and all-cause mortality within 12 months after discharge were identified through a review of the electronic medical record and administrative databases of the three participating hospitals.

### 2.5. Data Collection and Harmonization

For the CH-2005 cohort, data were collected retrospectively from discharge summaries and the original medical records using a standardized case report form (CRF) with predefined variable definitions (PREDICE framework). All data extractors received training to ensure that they consistently applied CRF definitions across centers. To keep the comparison with CH-2015 as fair as possible, we limited the analyses to variables that are routinely recorded in both periods (demographics, major comorbidities, whether echocardiography or natriuretic peptides were obtained, discharge medications, and hard outcomes).

### 2.6. Statistical Analysis

Quantitative variables were analyzed using Student’s *t*-test or the Mann–Whitney U test, depending on whether they showed a normal distribution. Differences in qualitative variables were analyzed using the χ^2^ test or Fisher’s exact test.

To reduce bias due to baseline differences between the 2005 and 2015 cohorts, a propensity score (PS) matching analysis was performed. PS was defined as the probability of belonging to the 2015 cohort based on the baseline covariates included in the logistic regression model: age, sex, and Charlson Comorbidity Index. Then, we applied 1:1 matching without replacement, obtaining a matched sample of 360 patients (180 per cohort). Covariate balance was assessed using standardized mean differences (SMDs) before and after matching. In the matched cohort, outcomes were compared by calculating the individual difference (2015 cohort–2005 cohort) for each matched pair and assessing whether the mean of these differences was non-zero using a one-sample *t*-test. Since the outcomes are binary, the mean difference was interpreted as an absolute risk difference (in percentage points), and point estimates, 95% confidence intervals, and *p*-values were reported.

The analyses were performed using the IBM SPSS Statistics 26 software package (IBM Corporation, Armonk, NY, USA).

## 3. Results

### 3.1. Demographic and Clinical Characteristics

The demographic and clinical characteristics of the two study cohorts are shown in Table 1. The mean age of the CH-2005 cohort was 73.6 years, and that of the CH-2015 cohort was 75 years (*p* = 0.16), with males accounting for 50.8% and 48.9% of the cohort, respectively (*p* = 0.64). Regarding comorbidities, the CH-2015 cohort had a higher proportion of patients with PAD and CKD and a lower proportion of individuals with IHD, valvular heart disease, and COPD, with the percentage of patients with a CCI > 2 being significantly higher (90% vs. 12.8%, respectively, *p* < 0.001). In the CH-2015 and CH-2005 populations, 0% and 22% had a CCI of 0 (no comorbidities), respectively.

### 3.2. Diagnostic Tests

The diagnostic test results of the two cohorts are shown in Table 2. TTE was performed more frequently in the CH-2015 cohort than the CH-2005 cohort (92.2% vs. 66.5%, *p* < 0.001), and the proportion of patients with a reduced LVEF was also higher in CH-2015 (37% vs. 26.6%, *p* < 0.001). No significant differences were found in NT-proBNP levels between the cohorts.

### 3.3. Treatment at Discharge

The treatments given at discharge to the two study cohorts are shown in Table 3. In CH-2015, the number of prescriptions for beta-blockers, statins, and oral anticoagulants at discharge was higher. The use of digoxin and nitrates was lower, while the prescription rates for the remaining evaluated drugs were similar between the two cohorts.

### 3.4. Readmissions and Mortality

The readmission and mortality rates of the two study cohorts are shown in Table 4. The CH-2015 cohort had a higher readmission rate at one month (35.6% vs. 7.3%, *p* < 0.001) and at one year (49.4% vs. 21.2%, *p* < 0.001), as well as a higher mortality rate (22.5% vs. 16.3%, *p* = 0.07).

### 3.5. Propensity Score (PS) Matching Analyses (Table 5 and Table 6)

The results from the PS matching analyses of the two study cohorts are shown in Table 5 and Table 6. After estimating the PS and implementing 1:1 matching without replacement, a matched sample of 360 patients (180 per cohort) was obtained. The two matched groups in the sample were comparable in terms of the baseline covariates included in the model (age, sex, and Charlson Comorbidity Index) (Table 5).

**Table 5:** Covariate baseline balance in the propensity score matched sample (*n* = 360, 1:1 matching).

	2005 Cohort (*n* = 180)	2015 Cohort (*n* = 180)	SMD
Age (years), mean ± SD	74.7 ± 9.6	75 ± 9.8	0.029
Male gender, *n* (%)	92 (51.1)	92 (51.1)	<0.001 *
Charlson Index, mean ± SD	2.8 ± 2.6	2.8 ± 2.9	0.026

SMD, standardized mean difference; SD, standard deviation. * For dichotomous variables, standardized mean differences were calculated by treating each category as a binary variable (1 = male, 2 = female).

**Table 6:** Outcomes in the propensity score-matched sample (*n* = 360, 1:1 matching).

	Absolute Risk Difference (2015–2005) *	95% CI	*p*
Heart failure readmission at one-month	0.267	0.184, 0.350	<0.001
Heart failure readmission at one-year	0.239	0.144, 0.334	<0.001
One-year mortality	0.067	−0.014, 0.147	0.1026

CI, confidence interval. * The difference corresponds to the mean of (2015–2005) and was tested using a one-sample *t*-test.

The outcomes in the matched cohorts are shown in Table 6. For readmissions after one month, the absolute risk difference was 0.267 (*p* < 0.001), which is equivalent to 26.7% more readmissions in 2015 compared to 2005 after balancing the cohorts in terms of age, sex, and comorbidities. For readmissions after one year, the absolute risk difference was 0.239 (*p* < 0.001), which is equivalent to 23.9% more readmissions in 2015 compared to 2005. For one-year mortality, the absolute risk difference was 0.067 (*p* = 0.1026), which is equivalent to 6.7% more readmissions in 2015 compared to 2005.

## 4. Discussion

### 4.1. Demographic and Clinical Characteristics

This study assessed the clinical characteristics of patients admitted for a first episode of HF in three university hospitals in Spain, the adherence to clinical practice guideline standards, and the one-year readmission and mortality rates. Data were collected from two patient cohorts separated by ten years (2005–2015), which allowed for the first direct evaluation of temporal changes in the management of early-stage HF in hospitalized patients in the European universal healthcare system.

Overall, the mean ages of the patients in both cohorts were similar and consistent with those reported in recent studies conducted in both population-based [13,14] and hospital-based HF patients [15,16] but higher than those from other studies that only included patients seen in Cardiology departments [17,18].

The significantly higher comorbidity observed in the CH-2015 cohort is striking (CCI > 2 in 90% vs. 13% in 2005). Considering the close association between CCI and disease prognosis [12], the high comorbidity burden in CH-2015 is likely the main factor responsible for the poorer clinical outcomes observed in this group. Although not shown in the Section 3, the proportion of patients with a CCI > 2 was significantly higher among those admitted to Internal Medicine compared to those admitted to Cardiology (27.8% vs. 18.8%, *p* = 0.034), findings that are consistent with previously published data [19,20]. This supports the key role of internists as integrative specialists managing multi-organ dysfunction and clinical frailty in hospitalized HF patients, a role that has already been highlighted in the literature [20,21,22].

In our study, a high prevalence of diabetes mellitus was observed, similar to that described in other studies conducted in Spain [14,18]. An increase in the frequency of patients with CKD and PAD over the study period was also noted, which has also been reported in previous studies [3,12,15,17,18]. Conversely, a decrease in the prevalence of ischemic heart disease and valvular heart disease was observed, which is consistent with the observed reduction in the frequency of HF with a reduced LVEF, both in our study and in other recent studies [2,23]. The relatively low prevalence of a reduced ejection fraction (HFrEF) in both cohorts is noteworthy and likely reflects the clinical heterogeneity of de novo acute heart failure in real-world hospital settings, where HFpEF is being increasingly recognized, especially among older patients with multiple comorbidities.

This worse clinical profile in the 2015 cohort can be explained by the comorbidity burden, which is the interrelation between multiple chronic diseases from different systems that frequently coexist and influence clinical outcomes, prognosis, and therapeutic responses. Unlike the traditional approach, which considers comorbidities in isolation, this perspective emphasizes the cumulative and synergistic effects of comorbidities as a complex network of pathophysiological interactions that weaken the patient’s functional reserve. In the present study, the increased prevalence of CKD, older age, and, above all, the functional decline observed in the CH-2015 cohort can be interpreted as an expression of this comorbidity burden.

An under-recognized phenotype that may contribute to de novo AHF presentations is tachycardia/arrhythmia-induced cardiomyopathy (tachycardiomyopathy), a potentially reversible form of LV systolic dysfunction driven by persistent tachyarrhythmias, frequently caused by atrial fibrillation. Contemporary evidence suggests that tachycardiomyopathy may account for a meaningful subset of HF admissions with a reduced LVEF, and that recurrence and long-term outcomes can vary depending on arrhythmia control and the underlying substrate defects [24,25]. In our cohorts, the substantial prevalence of atrial fibrillation raises the possibility that a proportion of the patients (particularly those with a reduced LVEF) could have had an arrhythmia-driven component, which may influence recovery trajectories and rehospitalization patterns over time.

### 4.2. Use of Diagnostic Tests

In our study, the proportion of patients who underwent echocardiography during hospitalization was higher in the CH-2015 cohort than in CH-2005 (92.2% vs. 66.5%, *p* < 0.001). These findings are similar to those reported in contemporaneous studies on CH-2005 [11,25] and CH-2015 [4,6]. Mean NT-proBNP values did not change over the study period and are also consistent with those reported in hospitalized patients in our setting [26].

### 4.3. Treatment at Discharge

The most notable finding was the significant increase in the prescription rate of beta-blockers at discharge between 2005 and 2015 (31.3% vs. 50.6%, respectively), indicating a clear improvement in the adherence to therapeutic guidelines over the study period. The patients given beta-blockers at discharge included those with HFpEF, likely reflecting attempts to control heart rate in the context of atrial fibrillation, treatment of coexisting ischemic heart disease, or hypertension rather than strict adherence to guideline-directed therapy for HFrEF. Regarding ACE inhibitors (ACEIs) and angiotensin II receptor blockers (ARBs), no substantial changes in their use were observed over the study period (69% in 2005 and 76.1% in 2015).

The prescription rates of these drugs are much lower than those reported in studies of ambulatory patients treated in Cardiology departments, such as the study by Crespo-Leiro et al. [18], where the beta-blockers prescription rate was 88.7% and the ACEI/ARB rate was 86.8%; however, the rates in this study are very similar to those found in other studies involving patients treated in Internal Medicine departments [27]. Possible reasons for the lower use observed in our study include the older age and higher comorbidity rate of the patients, the higher proportion of patients diagnosed with HF with preserved ejection fraction (HFpEF) (there is limited evidence supporting the use of these drugs [27,28,29]), and the fact that the study population comprised hospitalized patients in whom acute HF decompensation is often not fully resolved by the time of discharge, leading to initiation and titration of these treatments during follow-up visits. In this regard, the prescription rates in our study are consistent with those reported in other studies with a similar profile of hospitalized patients in Spain, such as the Spanish Registry of Acute Heart Failure (RICA) [30].

Additionally, it is worth noting that in our study, loop diuretics were the most frequently prescribed drugs in both cohorts, a finding that was also observed in previous studies [31,32].

### 4.4. Readmissions and Mortality

The natural history of HF is influenced by episodes of decompensation, which usually require hospitalization [33]. Worsening of HF diagnosed during the index episode causes up to 70% of readmissions [34].

In our study, despite improvements in the adherence to therapeutic guidelines between 2005 and 2015, there was a significant increase in readmission rates; this was more pronounced for one-month readmissions, which rose from 7.3% to 35.6%, but also present in one-year readmissions (21.2% vs. 49.4%). Regarding one-month readmissions, the rates observed in CH-2015 are much higher than those reported in other studies, such as that by Ko et al., which documented a 20.8% rate in Canadian patients during the 2006–2017 period [35].

Regarding one-year readmissions, figures from other contemporary studies on acute heart failure—both national [30] and international [36]—range between 24% and 25.6%, while for de novo acute HF in the Spanish Registry of Acute Heart Failure, the rate is 16%. These rates are similar to those seen in our CH-2005 cohort but are much lower than the rate in CH-2015.

Possible reasons for the higher percentage of readmissions in CH-2015 could include the greater comorbidity burden of this sample or differences in subject inclusion criteria, since most published studies are registry-based and typically do not include patients with limited life expectancy, whereas such patients were included in our study. Furthermore, our cohorts only included patients with de novo HF.

Regarding one-year mortality, our study also showed an increase that approaches statistical significance between 2005 and 2015, rising from 16.3% to 22.5%. In the literature, most contemporary studies report very similar one-year mortality rates. In the international GREAT registry, the rate was 20% for the entire cohort [37,38,39] and 32% for European patients [40]; in a registry of patients admitted to Cardiology departments between 2013 and 2014, the rate was 20.5% [16]; and in the RICA registry, it was 23% for acute HF patients and 15% for those with de novo acute HF [30].

However, the patients in the 2015 cohort had a greater comorbidity burden than those in the 2005 cohort (mean Charlson Index: 2.9 ± 2.9 vs. 2.2 ± 2.4; *p* < 0.001). To improve comparability of outcomes between the 2005 and 2015 cohorts, a propensity score (PS) matching analysis was performed. The balance between the matched cohorts in terms of the covariates included in the model (age, sex, and Charlson Comorbidity Index) was excellent. In the propensity matching analysis, readmissions at one month and one year in the 2015 cohort were significantly higher, and no significant differences were observed in mortality at one year.

### 4.5. Limitations

The main limitation in this study is the comparison of a retrospective and a prospective study, which may limit the scope of the conclusions drawn, particularly regarding the differences in diagnostic and therapeutic management.

Although the analysis of the CH-2005 data used a standardized abstraction protocol and trained extractors, temporal changes in medical record completeness and diagnostic availability over the decade could have still introduced differential information bias (e.g., under- or over-estimation of comorbidities and testing). To mitigate this, we focused the comparisons on routinely recorded, objective data elements and excluded cases in which HF was not confirmed at discharge. Nevertheless, residual information bias related to evolving documentation practices cannot be fully excluded. Despite the standardization efforts made, we also recognize that the retrospective design of the CH-2005 cohort analysis could have some degree of information bias due to the less structured data and variable quality in medical records from that period. Therefore, minor differences in the granularity or completeness of certain variables (e.g., laboratory testing or treatment) cannot be fully ruled out. Furthermore, the smaller size of the 2015 cohort may have limited the power to detect small or moderate differences in a relatively rare outcome such as mortality.

PS matching was limited to baseline variables that both cohorts had data for (age, sex, and comorbidity burden), and residual confounding cannot be excluded.

A limitation of the CH-2015 study, which is inherent to unblinded prospective designs, is the awareness of healthcare professionals that their clinical practice is being evaluated (Hawthorne effect), which may have led to greater adherence to guidelines, especially in relation to diagnostic testing and potentially discharge treatment. Both of these factors were always left to the discretion of the attending physician.

Information on prior myocardial infarction and coronary revascularization was not systematically captured as predefined variables in both cohorts; therefore, etiologic granularity regarding ischemic substrate is limited.

Finally, another potential limitation—derived from the multicenter nature of the study—is the variability among data extractors. This was minimized through the development of an explicit data collection protocol and training in specific workshops.

### 4.6. Clinical Implications and Future Outlook

The changes observed in our study have high relevance in light of recent developments and advances in the understanding and treatment of HF. The most recent clinical practice guidelines [28,41,42] have redefined the clinical phenotypes of this condition, recognizing the association of HF with preserved ejection fraction with advanced age, comorbidities, and frailty [28,41,42].

Likewise, the therapeutic approach based on the “four pillars”—beta-blockers, ACEIs/ARBs or sacubitril-valsartan, mineralocorticoid receptor antagonists, and sodium-glucose cotransporter 2 inhibitors—has become consolidated, particularly in patients with HF and a reduced ejection fraction. Although these treatments were not part of the therapeutic arsenal available during the observation period of our study, their current use must be taken into account when interpreting the results of longitudinal studies such as this one. Moreover, they underscore the need for organizational strategies that ensure their implementation in complex clinical settings such as Internal Medicine departments, where the typical patient presents with multiple comorbidities, advanced age, and significant functional frailty.

The findings of this study, although conducted within the Spanish healthcare system, reflect a broader clinical phenomenon observed across the European continent. The transition toward an older, more complex patient profile with a high burden of multimorbidity is a hallmark of the contemporary European comorbidity burden. Our data align with global trends [43], suggesting that the management of de novo acute heart failure in the elderly is no longer a purely cardiological issue but a systemic challenge that requires the integrative approach typical of European Internal Medicine departments [20]. By framing heart failure within this universal European context of aging populations, our results emphasize the urgent need for organizational strategies that address the prognostic impact of clinical frailty and multi-organ dysfunction, as highlighted in recent clinical updates [42].

## 5. Conclusions

Over a decade, the profile of de novo acute HF transitioned toward an older, multimorbid phenotype with worsening post-discharge outcomes despite improved guideline adherence. These findings suggest that HF management must evolve from a cardiac-centric approach toward an integrated, multi-organ strategy led by specialists capable of managing clinical frailty and complex comorbidity burdens.

## Figures and Tables

**Table 1:** Demographic and clinical characteristics and functional status of patients in CH-2005 and CH-2015 cohorts from the PREDICE study.

	2005 Cohort (*n* = 600)	2015 Cohort (*n* = 180)	*p*
Age (years), mean ± SD	73.6 ± 12.3	75 ± 9.7	0.16
Male gender, *n* (%)	305 (50.8)	88 (48.9)	0.64
Heart diseases			
Ischemic heart disease, *n* (%)	98 (16.3)	18 (10.1)	0.04
Valvular heart disease, *n* (%)	160 (26.7)	23 (15)	<0.001
Atrial fibrillation, *n* (%)	177 (29.5)	56 (31.1)	0.67
Comorbidities			
Diabetes mellitus, *n* (%)	246 (41)	79 (43.9)	0.49
Peripheral vascular disease, *n* (%)	65 (10.8)	26 (14.4)	0.08
COPD, *n* (%)	112 (18.7)	25 (13.9)	0.07
Chronic kidney disease, *n* (%)	71 (11.8)	32 (17.8)	0.01
Functional independence			
Charlson Index > 2	77 (12.8)	162 (90)	<0.001
Independent in activities of daily living, *n* (%)	537 (89.5)	160 (88.9)	0.82

SD, standard deviation; COPD, chronic obstructive pulmonary disease.

**Table 2:** Diagnostic tests performed during hospitalization on patients included in CH-2005 and CH-2015 from the PREDICE study.

	2005 Cohort (*n* = 600)	2015 Cohort (*n* = 180)	*p*
Transthoracic echocardiography during hospitalization			
Number performed, *n* (%)	399 (66.5)	166 (92.2)	<0.001
LVEF < 40%, *n* (%)	106 (26.6)	54 (37)	<0.001
Laboratory test results			
Hemoglobin [g/L], mean ± SD	12.8 ± 2.3	12.3 ± 2.2	0.01
Plasma sodium [mEq/L], mean ± SD	138.1 ± 4.6	138.5 ± 5	0.32
Plasma potassium [mEq/L], mean ± SD	4.3 ± 0.7	4.4 ± 0.7	0.09
eGFR [mL/min], mean ± SD	75.0 ± 38.6	72.0 ± 30.7	0.63
NT-pro-BNP [pg/mL], mean ± SD	3680.2 ± 3610.2	3955.5 ± 4944.3	0.41

LVEF, left ventricular ejection fraction; SD, standard deviation; eGFR, estimated glomerular filtration rate; NT-proBNP, N-terminal fragment of B-type natriuretic peptide.

**Table 3:** Discharge treatment for patients included in CH-2005 and CH-2015 from the PREDICE study.

	2005 Cohort (*n* = 600)	2015 Cohort (*n* = 180)	*p*
Beta-blockers, *n* (%)	188 (31.3)	90 (50.6)	<0.001
ACE inhibitors, *n* (%)	328 (54.7)	96 (53.9)	0.75
ARBs, *n* (%)	104 (17.3)	27 (15.3)	0.46
ACE inhibitors—ARBs, *n* (%)	414 (69.0)	137 (76.1)	0.114
MRA, *n* (%)	104 (17.3)	27 (15.3)	0.46
Loop diuretics, *n* (%)	439 (73.2)	137 (77)	0.43
Digoxin, *n* (%)	152 (25.3)	26 (14.6)	0.002
Nitrates, *n* (%)	70 (11.7)	8 (4.9)	0.005
Antiplatelets, *n* (%)	263 (43.8)	83 (46.6)	0.59
Oral anticoagulants, *n* (%)	203 (33.8)	75 (42.1)	0.05
Statins, *n* (%)	150 (25)	86 (48.3)	<0.001

ACE, angiotensin-converting enzyme; ARBs, angiotensin receptor blockers; MRAs, mineralocorticoid receptor antagonists.

**Table 4:** One-year outcomes in patients included in CH-2005 and CH-2015 from the PREDICE study.

	2005 Cohort (*n* = 600)	2015 Cohort (*n* = 180)	*p*
Heart failure readmission at 1 month, *n* (%)	44 (7.3)	64 (35.6)	<0.001
Heart failure readmission at 1 year, *n* (%)	127 (21.2)	89 (49.4)	<0.001
One-year mortality, *n* (%)	98 (16.3)	40 (22.5)	0.07

## Data Availability

The datasets used and analyzed in the study are available from the corresponding author upon reasonable request.

## Abbreviations

The following abbreviations are used in this manuscript:

ACEIs	Angiotensin-converting enzyme inhibitors
AHF	Acute heart failure
ARBs	Angiotensin receptor blockers
CCI	Charlson Comorbidity Index
CH-2005	Retrospective 2005 cohort
CH-2015	Prospective 2015 cohort
CKD	Chronic kidney disease
COPD	Chronic obstructive pulmonary disease
CPGs	Clinical practice guidelines
DM	Diabetes mellitus
eGFR	Estimated glomerular filtration rate
HF	Heart failure
HFpEF	Heart failure with preserved ejection fraction
HFrEF	Heart failure with reduced ejection fraction
IHD	Ischemic heart disease
LVEF	Left ventricular ejection fraction
MRA	Mineralocorticoid receptor antagonists
NT-proBNP	N-terminal pro-B-type natriuretic peptide
PAD	Peripheral arterial disease
PS	Propensity score
SMD	Standardized mean differences
TTE	Transthoracic echocardiography
